# The *Caenorhabditis* RNA-seq Browser: a web-based application for on-demand analysis of publicly available *Caenorhabditis* spp. bulk RNA-sequencing data.

**DOI:** 10.17912/micropub.biology.001208

**Published:** 2024-05-07

**Authors:** Damia Akimori, LaDeana W. Hillier, Astra S. Bryant

**Affiliations:** 1 Department of Microbiology, Immunology, and Molecular Genetics, University of California, Los Angeles, Los Angeles, California, United States; 2 Molecular Biology Interdepartmental Ph.D. Program, University of California, Los Angeles, Los Angeles, California, United States; 3 Department of Genome Sciences, University of Washington, Seattle, Washington, United States; 4 Department of Physiology and Biophysics, University of Washington, Seattle, Washington, United States

## Abstract

The *Caenorhabditis* RNA-seq Browser is an open-source Shiny web app that enables on-demand visualization and quantification of bulk RNA-sequencing data for five *Caenorhabditis* species: *C. elegans* , *C. briggsae* , *C. brenneri* , *C. japonica* , and *C. remanei* . The app is designed to allow researchers without previous coding experience to interactively explore publicly available *Caenorhabditis* RNA-sequencing data. Key app features include the ability to plot gene expression across life stages for user-specified gene sets, and modules for performing differential gene expression analyses. The *Caenorhabditis* RNA-seq Browser can be accessed online via shinyapps.io or can be installed locally in R from a GitHub repository.

**Figure 1. Diagram of the Caenorhabditis RNA-seq Browser workflow and examples of the Shiny user interface:**
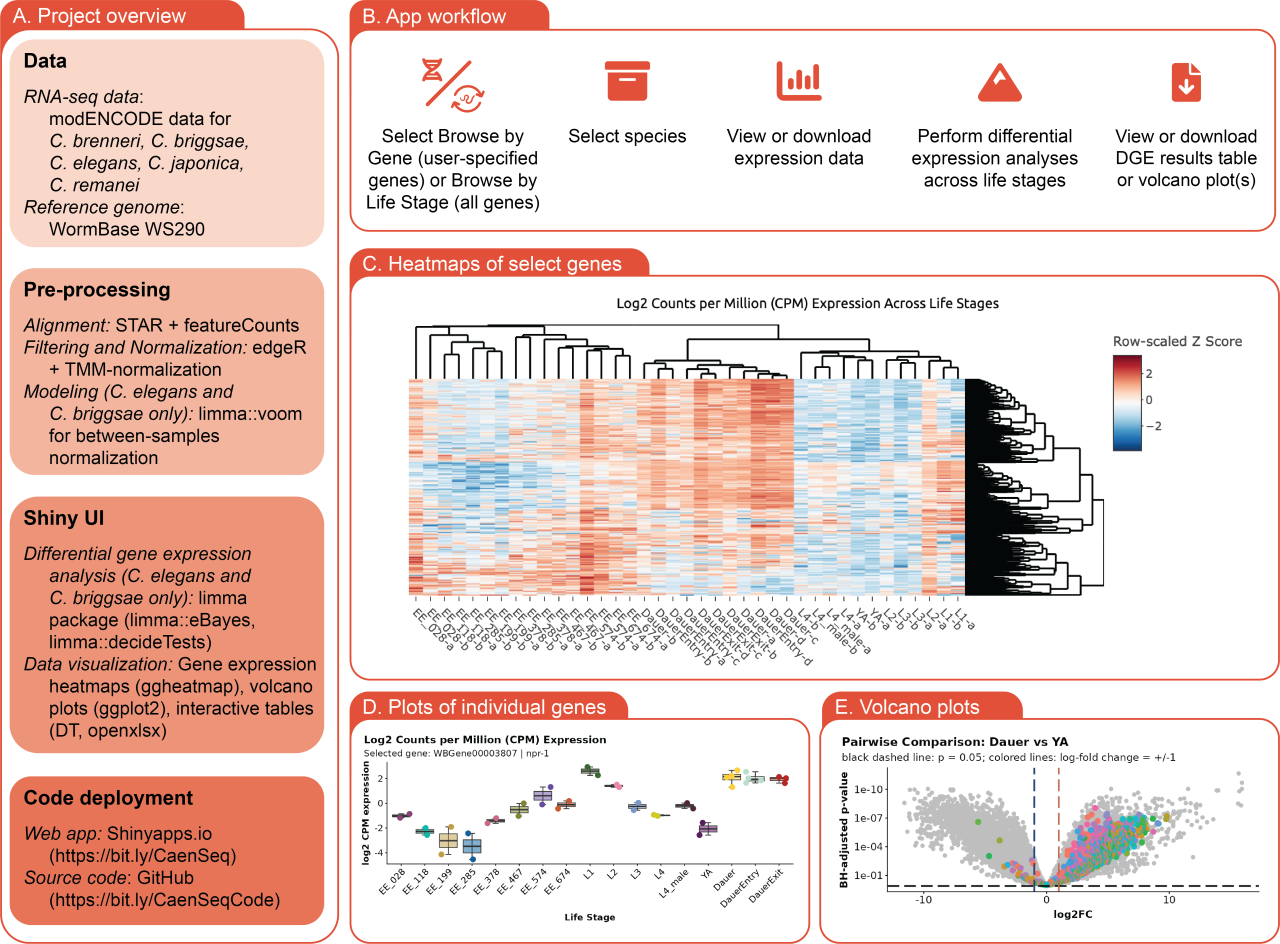
**A)** Overview of this project, including the data included, our pre-processing workflow, features of the Shiny user interface, and deployment information. **B)** User workflow for the *Caenorhabditis* RNA-seq Browser. **C)** Heatmap generated by the app in "Browse by Gene" mode. Search term used to generate the image was “GPCR”; species is *C. elegans* . Heatmap rows reflect log _2_ CPM values of individual genes and are ordered using Pearson clustering; columns reflect individual samples (life stages) are ordered by Spearman clustering. **D)** Plots of log _2_ CPM values for an individual gene ( *C. elegans npr-1* ) across life stages. **E)** Volcano plot of differential gene expression comparing *C. elegans* dauers and young adults.

## Description

**1. Introduction**

The free-living bacterivore *Caenorhabditis elegans* is a broadly used genetic model species. The enduring popularity of *C. elegans* is reflected in a wealth of publicly available genomic data, including a highly curated reference genome that is freely distributed via the online repository WormBase (Davis et al., 2022; Howe et al., 2016) . Furthermore, *C. elegans* has been included in a number of large-scale genomics projects, including the Model Organism Encyclopedia of DNA Elements (modENCODE), a consortium-based effort to identify a comprehensive list of functional genome elements in *C. elegans* and *Drosophila melanogaster* (Celniker et al., 2009; Gerstein et al., 2010, 2014) *.* A growing interest in comparative genomics within the phylum Nematoda has motivated the development of several other *Caenorhabditis* species as emerging genetic model organisms (Gerstein et al., 2014; Moya et al., 2023) . WormBase hosts high-quality reference genomes for multiple *Caenorhabditis* species, and ongoing efforts are continually improving the quality of reference annotations and functional genomics toolkits in these species (Davis et al., 2022; Moya et al., 2023) . Studies of the transcriptome in *C. elegans* and other *Caenorhabditis* species, as well RNA resources produced by the modENCODE project, have resulted in an abundance of publicly available bulk and single-cell RNA-sequencing datasets.

Multiple web-based tools are available for researchers seeking to explore *C. elegans* single-cell RNA-sequencing (RNA-seq) data, including the CeNGENApp and the WormBase-hosted *scdefg* and *wormcells-viz* (Da Veiga Beltrame et al., 2022; Davis et al., 2022; Hammarlund et al., 2018) . Together, these apps enable users to visualize gene expression across cell types and perform differential expression analyses of single-cell RNA-seq data (Da Veiga Beltrame et al., 2022; Hammarlund et al., 2018) . For bulk RNA-seq data, both WormBase and the CeNGENApp give users access to individual gene abundance in some *Caenorhabditis* species (Davis et al., 2022; Hammarlund et al., 2018) . However, researchers seeking to visualize expression across multiple genes or to quantify differential expression across developmental life stages must generate these analyses themselves, thus requiring computational resources and a degree of bioinformatics expertise. To enable researchers without previous coding experience to perform on-demand explorations of *Caenorhabditis* spp. bulk RNA-seq datasets, we have developed the *Caenorhabditis* RNA-seq Browser ( Fig. 1A ). This R-based tool features modules for visualization and quantification of differential gene expression across life stages in a set of five *Caenorhabditis* species: *C. elegans* , *C. brenneri* , *C. briggsae* , *C. japonica* , and *C. remanei* . For *C. elegans* , the app features an embryonic developmental timeline dataset as well as post-embryonic life stages. Source code for this tool is publicly available on GitHub at https://bit.ly/CaenSeqCode ; a web-based version is deployed at https://bit.ly/CaenSeq . Ultimately, we hope that this interactive application will serve as an open-source, user-friendly portal for accessing and analyzing *Caenorhabditis* spp. genomic expression data.

**2. Description of the *Caenorhabditis* RNA-seq Browser**

*2.1 Data sets.* The data included in the *Caenorhabditis* RNA-seq Browser are bulk RNA-seq datasets, including several that were previously published by the Waterston Lab (University of Washington) and/or collected as part of the modENCODE project (Boeck et al., 2016; Gerstein et al., 2010, 2014; Warner et al., 2019) . Information about RNA-seq experimental procedures, worm strains, and individual samples are available as study design files in our GitHub repository. In brief, the following samples are included in the app:

*C. brenneri* : early embryonic, L4, adult female, adult male.

*C. japonica* : early embryonic, L2, L4 adult female, adult male.

*C. remanei* : early embryonic, L2, L4, young adult female, young adult male.

*C. briggsae* : early embryonic, L2, L4, adults, mixed stages.

*C. elegans* : L1, L2, L3, L4, L4 males, young adults, dauer, dauer entry, dauer exit, and an embryonic timeline. The *C. elegans* embryonic samples reflect a unified timeline that was previously generated by inferring biological replicates across four independently collected embryo time series (Boeck et al., 2016) . Thus, embryo group names displayed in the app represent the average time for biological replicate pairs, which were selected to represent 80-minute developmental intervals.

*2.2 Alignment.* Genomes and GFF files were downloaded from WormBase version WS290. Reads were aligned to each genome using STAR (v2.7.6a, --alignIntronMax 30000 --alignMatesGapMax 30000) and the species-specific WS290 GFF file for each genome. PCR duplicates were removed using the SelDup command (Warner et al., 2019) . Read counts were obtained for each gene (CDS region only, which is labeled as "CDS" in *C. briggsae* and *C. elegans* and as "coding_exon" for *C. remanei* , *C. japonica* , and *C. brenneri* ) using the featureCounts function in the subread v2.0.6 software package, using default settings. Only uniquely mapping reads were counted.

*2.3 Filtering and normalization.* Filtering and normalization of read counts was performed in R as previously described (Bryant et al., 2021) . In brief, aligned read data for each species was imported into R v4.3.2 and annotated with gene information downloaded via WormBase ParaSite BiomaRT. Raw reads were quantified as counts per million (CPM) using the EdgeR package v4.0.2, then filtered to remove transcripts with low counts (Robinson et al., 2010) . For *C. elegans* and *C. briggsae* , the cutoff was ≥ 1 CPM in at least two samples; for all other species the cutoff was ≥ 1 CPM in at least one sample. Filtered counts were normalized using the trimmed mean of M-values (TMM) method (Robinson & Oshlack, 2010) . *C. elegans* and *C. briggsae* data are additionally processed for in-app differential gene expression analyses. For these species, the mean-variance relationship was modeled using a precision weights approach using the voom function in the limma package v3.58.1 (Ritchie et al., 2015) . A design matrix for comparisons across samples was generated for in-app linear modeling. For other species, the lack of consistent biological replicates precluded processing with limma::voom; we therefore chose to only enable app-based visualization of filtered and normalized gene count data.

*2.4 In-app differential gene expression analyses.* For *C. elegans* and *C. briggsae* data, user-defined pairwise differential expression analyses are performed as previously described (Bryant et al., 2021) . In brief, variance-stabilized, filtered, normalized log _2_ CPM data are fitted to a linear model using the limma::lmFit function and the pre-generated design matrix. The limma::eBayes function is used for empirical Bayes smoothing of gene-wise standard deviations (Phipson et al., 2016; Smyth, 2004) . Differentially expressed genes are identified using the limma::decideTests function, with *p* -values adjusted for multiple comparisons using the Benjamini-Hochberg false discovery rate method (Benjamini & Hochberg, 1995; Ritchie et al., 2015) . Users may choose to correct for multiple pairwise comparisons. Genes with statistically significant changes of gene expression are defined as those with a false discovery rate of ≤ 0.05 and an absolute log _2_ fold change of ≥ 1.

*2.5 Shiny user interface* . The interactive application was created in R using the Shiny package v1.8.0, as previously described (Bryant et al., 2021) . Data visualizations are performed using the following functions/packages: heatmaply v1.5.0, dendextend v1.17.1, ggplot2 v3.4.4, DT v0.3.0, openxlsx v 4.2.5.2, and stats v4.3.2 (Galili et al., 2018; Wickham, 2009) .

The app is designed to enable an interactive workflow where users are guided through data visualization and analysis by a sequence of graphical user interfaces ( Figure 1B ). Users first must pick between interfaces allowing comparisons of selected genes (Browse by Gene tab) or across all genes (Browse by Life Stage tab). Users then select a species of interest using a dropdown menu. All five *Caenorhabditis* species are available for selection in the Browse by Gene tab; only *C. elegans* and *C. briggsae* are available in the Browse by Life Stage tab. In the Browse by Gene tab, users are prompted to input genes of interest either as gene names, stable IDs, or keywords that are matched to InterPro gene descriptions. If users submit multiple genes of interest, the app displays a heatmap of gene expression across life stages, with samples (life stages) ordered by Spearman clustering and genes ordered using Pearson clustering ( Figure 1C ). Users may use a dropdown menu to view gene expression plots for individual genes as well as a data table containing log _2_ CPM data and gene annotation information for all submitted genes ( Figure 1D ). Plots and data tables can be downloaded as editable PDFs or Microsoft Excel spreadsheets, respectively. For *C. elegans* and *C. briggsae* , users are then prompted to select pair-wise comparisons across life stages for differential expression analyses; users may either use a dropdown menu to specify a single comparison or a textbox to set multiple comparisons. In the Browse by Life Stage tab, user input of the desired pair-wise comparisons occurs immediately after species selection. After users specify the desired comparisons, the app generates volcano plots and a data table that displays log _2_ CPM data, gene annotation information, and differential expression analysis results ( *e.g.,* logFC, Benjamini-Hochberg-adjusted *p* -values; Figure 1E ). Volcano plots are downloadable as editable PDFs; users seeking to download the differential expression results data tables as Microsoft Excel spreadsheets can first filter the data if desired ( *e.g.* , to save only downregulated genes, or the top 10% of upregulated genes). For all species, users may download the study designs as .txt files and the log _2_ CPM data generated during preprocessing as either .csv files or R objects.

**3. Conclusion**

The *Caenorhabditis* RNA-seq Browser is a Shiny web application that aims to provide a user-friendly software resource for nematode researchers seeking to explore and quantify gene expression across developmental life stages. This software is inspired by the *Strongyloides* RNA-seq Browser and shares core functionality and an updated code infrastructure (Bryant et al., 2021) . Ultimately, we hope that the *Caenorhabditis* RNA-seq Browser will help scientists without previous coding experience utilize the wealth of published RNA-seq data for *Caenorhabditis* species in their research.
